# The Peptide Specificity of the Endogenous T Follicular Helper Cell Repertoire Generated after Protein Immunization

**DOI:** 10.1371/journal.pone.0046952

**Published:** 2012-10-15

**Authors:** Scott A. Leddon, Andrea J. Sant

**Affiliations:** David H. Smith Center for Vaccine Biology and Immunology, Department of Microbiology and Immunology, University of Rochester Medical Center, Rochester, New York, United States of America; MRC National Institute for Medical Research, United Kingdom

## Abstract

T follicular helper (Tfh) cells potentiate high-affinity, class-switched antibody responses, the predominant correlate of protection from vaccines. Despite intense interest in understanding both the generation and effector functions of this lineage, little is known about the epitope specificity of Tfh cells generated during polyclonal responses. To date, studies of peptide-specific Tfh cells have relied on either the transfer of TcR transgenic cells or use of peptide∶MHC class II tetramers and antibodies to stain TcR and follow limited peptide specificities. In order to comprehensively evaluate polyclonal responses generated from the natural endogenous TcR repertoire, we developed a sorting strategy to separate Tfh cells from non-Tfh cells and found that their epitope-specific responses could be tracked with cytokine-specific ELISPOT assays. The immunodominance hierarchies of Tfh and non-Tfh cells generated in response to immunization with several unrelated protein antigens were remarkably similar. Additionally, increasing the kinetic stability of peptide-MHC class II complexes enhanced the priming of both Tfh and conventional CD4 T cells. These findings may provide us with a strategy to rationally and selectively modulate epitope-specific Tfh responses. By understanding the parameters that control epitope-specific priming, vaccines may be tailored to enhance or focus Tfh responses to facilitate optimal B cell responses.

## Introduction

The generation of a high-affinity class-switched antibody response is the most common benchmark for successful vaccination (reviewed in [1], [2]). T follicular helper (Tfh) cells are an indispensable and limiting factor during the germinal center response [3]–[5] that gives rise to both memory B cells and long-lived plasma cells, which in turn generate and sustain protective antibody responses (reviewed [6], [7]). While much progress has been made in understanding the development and function of the Tfh lineage over the past several years, questions about the diversity and peptide specificity of the Tfh response generated after immunization remain unaddressed.

After immunization or infection, naïve T cells are initially primed through interaction with antigen-bearing dendritic cells (DC) in the T cell zone. As a consequence of interactions with DC, a fraction of the activated T cells gain expression of CXCR5 and BCL6 and decrease CCR7 expression. This change in chemokine receptor expression allows for migration of these T cells from the T cell zone to the border of the B cell zone and also into the interfollicular zones [8]–[10]. Here, they have an opportunity to interact with peptide-presenting B cells prior to entry into germinal centers. Cognate antigen presentation by germinal center B cells is required to recruit T cell help for class-switching, affinity maturation, and differentiation into memory and long-lived plasma cells (reviewed in [11]). While it is clear that DC are necessary and sufficient for the initiation of the Tfh response [12]–[14], several experimental systems in which B cells are either absent [14]–[17], deficient in MHC class II gene expression [13], or are incapable of sustained interactions with T cells [18]–[20] have shown that B cells and B cell antigen presentation are required for sustaining the Tfh response beyond the first few days of the immune response (reviewed in [21]–[27]), accumulation of Tfh cells within the B cell follicles, and for Tfh cells to express high levels of the effector molecules PD-1 and IL-21 [12], [28].

Because cognate interactions are required for T cell priming and Tfh differentiation, the sets of peptides presented by DC and B cells are likely to influence the specificity of Tfh cells generated during an immune response. Differences in how B cells and DC access, acquire, process, and edit antigen could result in these cell types presenting distinct repertoires of peptide-MHC class II complexes [29]–[33] (reviewed in [34]–[39]). If B cells are unable to present epitopes that are presented by DC during initial priming, after the first few days of the immune response T cells specific for these epitopes will not be retained in the Tfh pathway and thus will not participate in germinal center reactions. Therefore, the specificity of Tfh cells after the first few days of the immune response may represent a functional readout of B cell antigen presentation *in vivo*.

For this study, an experimental system was developed to evaluate the peptide specificity of endogenous polyclonal Tfh cells after protein immunization. Responses were evaluated in BALB/c mice, which express both I-A^d^ and I-E^d^ MHC class II molecules, because the CD4 T cell immunodominance hierarchy has been well characterized for multiple model antigens [40]–[47]. IL-21, IL-4, IL-2, and IFNγ cytokine-specific ELISPOT assays were used to evaluate the frequency and diversity of the peptide-specific Tfh and non-Tfh responses generated after immunization with several model antigens. This study also evaluated the impact of increasing or decreasing the kinetic stability of peptide-MHC class II complexes on the elicitation of Tfh cells *in vivo*. To our knowledge, this is the first report of the antigen specificity of Tfh cells generated during an endogenous polyclonal response.

## Materials and Methods

### Ethics Statement

All experiments for this study were performed in strict accordance with the recommendations in the Guide for the Care and Use of Laboratory Animals of the National Institutes of Health and with the approval of Animal Care and Use Committees at the University of Rochester (protocol 2008-023).

### Protein Production and Purification

Purified hen egg lysozyme (HEL) and chicken ovalbumin (OVA) were purchased from Sigma-Aldrich (St. Louis, MO). Production and purification of maltose binding protein from *E. coli* (MalE) was previously described [48].

### Immunizations

Two to four month old BALB/c mice (National Cancer Institute, Frederick, MD) were immunized in the pinna of both ears with 10 µL of an IFA/PBS emulsion containing 5 µg of protein (10 µg/mouse) and 0.6 µg/mL LPS (Sigma-Aldrich). For experiments evaluating kinetic stability peptide variants, the portion of the pinna containing the emulsion was excised three days post-immunization. For cell sorting experiments, unless otherwise indicated, for each immunizing protein and replicate experiment, 50–70 mice were sacrificed 8 to 9 days post-immunization, and draining cervical lymph nodes were harvested and pooled as the source of T cells for assays.

### Antibodies and Peptides

Purified anti-IL-2 (JES6-1A12), anti-IL-2-biotin (JES6-5H4), purified anti-IFNγ (AN-18), anti-IFNγ-biotin (XMG1.2), purified anti-IL-4 (11B11), anti-IL-4-biotin (BVD6-24G2), Fc Block (2.4G2), anti-CD4-PE-Cy7 (RM4-5), anti-CD4-V450 (RM4-5), anti-B220-PE-Cy5 (RA3-6B2), anti-CD44-APC-Cy7 (IM7), anti-CXCR5-biotin (2G8), and anti-BCL6-Alexa647 (K112-91) antibodies were obtained from BD Biosciences (San Jose, CA). Anti-ICOS-Alexa488 (C398.4A), anti-CD69-Alexa488 (H1.2F3), anti-CD62L-Alexa488 (MEL-14), and anti-CCR7-Alexa488 (4B12) antibodies were obtained from BioLegend (San Diego, CA). Anti-PD1-FITC (J43), anti-PD1-PE-Cy7 (J43), and IL-21 ELISPOT capture and detection (components of the Mouse IL-21 ELISPOT Ready-SET-Go! reagent set) antibodies were obtained from eBioscience. All synthetic peptides were purchased from BioPeptide (San Diego, CA) or produced in-house as previously described [49].

### Analytical Cell Staining and Flow Cytometry

**Figure 1:**
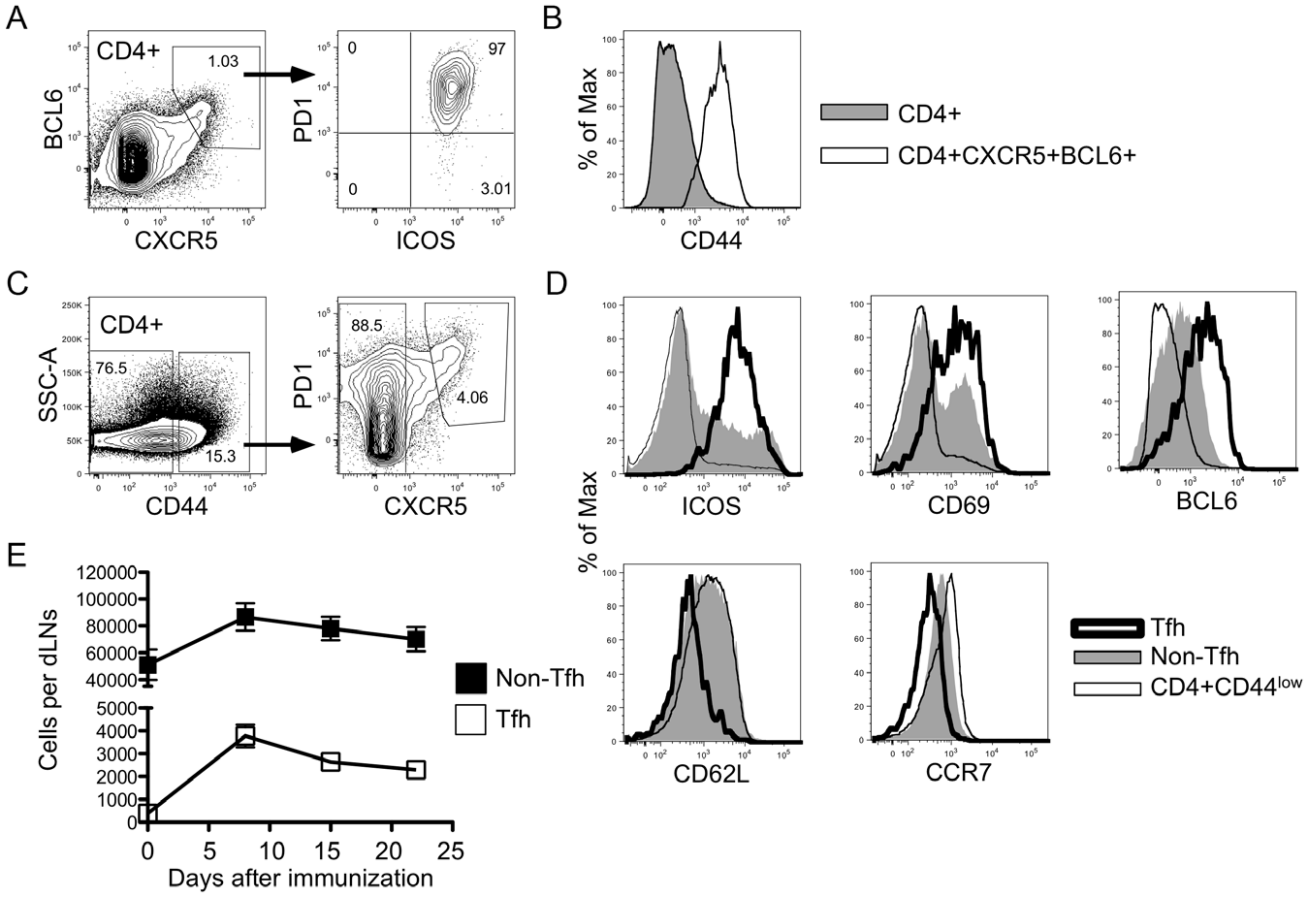
The conical phenotypic markers of Tfh cells established in other mouse strains are maintained in BALB/c mice. Animals were immunized subcutaneously in the pinna of the ear with MalE protein emulsified in IFA. Nine days later, CD4+ cells from draining cervical lymph nodes were analyzed for Tfh cell phenotypic markers. (A) CD4+CXCR5^high^BCL6+ cells were analyzed for dual expression of PD1 and ICOS. (B) CD44 staining of CD4+ (filled) and CD4+CXCR5^high^BCL6+ (open) cells. (C) Gating strategy gating of Tfh, non-Tfh, and CD4+CD44^low^ cell populations represented in D and E. (D) Expression of ICOS, CD69, BCL6, CD62L, and CCR7 are shown for Tfh (thick line), non-Tfh (shaded), and CD4+CD44^low^ (thin line) populations. (E) Absolute numbers of Tfh (open squares) and non-Tfh (closed squares) cells within the lymph nodes were calculated by multiplying the percent of each cell type by the total number of cells from the harvested lymph nodes. Error bars show the standard error of the mean (S.E.M) of three to five single mouse experiments per time point.

Single cell suspensions were prepared and depleted of red blood cells (RBC) by treatment with ACK lysis buffer (0.15M NH_4_Cl/1 mM KHCO_3_/0.1 mM Na_2_-EDTA in H_2_O, pH 7.2). Cells were then stained with Live/Dead Fixable Aqua Dead Cell Stain Kit (Life Technologies, Carlsbad, CA). After vital staining, cells were preincubated with Fc Block (BD Biosciences) before the addition of surface staining antibodies. After incubation with mAbs at 4°C for 30 minutes, cells were washed and incubated with PE-streptavidin to detect anti-CXCR5 mAb binding. Prior to BCL6 staining, cells were fixed and permeabilized with BD Pharmingen Transcription Factor Buffer Set (BD Biosciences). Data were collected on a FACSCanto flow cytometer (BD Biosciences), and analyzed with FlowJo 8.6 software (Tree Star, Inc., Ashland, OR). All events shown in Figure 1 were pre-gated on B220-CD4+Live/Dead negative (live), with doublets excluded.

### Preparative Staining and Cell Purification

Single cell suspensions from the cervical lymph nodes of immunized mice were prepared and depleted of RBC by treatment with ACK lysis buffer (0.15M NH_4_Cl/1 mM KHCO_3_/0.1 mM Na_2_-EDTA in H_2_O, pH 7.2). Cell suspensions were then depleted of non-CD4 T cells by use of negative paramagnetic bead selection (CD4 T Cell Isolation Kit II, Miltenyi Biotec, Auburn, CA). For Treg depletion, CD25+ cells were depleted through the use of negative paramagnetic bead selection (CD4+CD25+ Regulatory T cell Isolation Kit, Miltenyi Biotec). For Tfh sorting, CD4-enriched cells were incubated with Fc Block (BD Biosciences) before the addition of anti-CD4, -CD44, -CXCR5, and -PD1 mAbs. After surface staining (4°C for 30 minutes), cells were washed and incubated with streptavidin-PE to detect anti-CXCR5 mAb staining. Cell sorting was preformed using a FACSAria (BD Biosciences), to isolate CD4+CD44^high^CXCR5^high^PD1^high^ Tfh cells and CD4+CD44^high^ cells that did not express high levels of CXCR5 or PD1 (non-Tfh cells). The purity of sorted populations was typically 90–95%. Splenocytes from naïve mice were depleted of RBC by treatment with ACK lysis buffer and were used as a source of APC in ELISPOT assays. The isolation of cells from the ear derma was performed as previously described [50].

### Cytokine-specific CD4 T cell ELISPOT Assay

Cytokine-secreting T cells were quantified at 18 h (IL-2, IL-4, and IFNγ) or at 40 h (IL-21) with cytokine-specific ELISPOT assay as described previously [45], [48]. In all cases 10 µM of peptide in the presence of syngeneic APC were used to stimulate T cells, which were added at densities that allowed readable spot counts in each well. Cytokine-specific spots were enumerated with an Immunospot Reader Series 2A (Immunospot Software V.3.2, CTL, Shaker Heights, OH). To determine the frequency of peptide-specific spots, spot counts from background wells (spots measured in the absence of peptide) were subtracted from wells with peptide and normalized to peptide-specific spots per 1,000,000 input CD4 T cells. In all cases, the number of peptide-specific cells was at least 2-fold over background. The frequencies of peptide-specific spots were summed to determine total cytokine-specific spots. The fractional response of each peptide was determined by calculating the percent of the total peptide-specific spots represented by each peptide epitope.

### Transcriptional Analysis

Total RNA was purified from sorted cells was extracted with TRIzol (Life Technologies, Carlsbad, CA) and used to synthesize cDNA (Ovation PicoSL WTA Kit, NuGEN Technologies, San Carlos, CA). TaqMan Universal PCR Master Mix, No AmpErase and the following TaqMan Gene Expression FAM dye-labeled, non-primer limited assays were obtained from Life Technologies: *Bact* (Mm00607939_s1), *Bcl6* (mm00477633_m1), *B2m* (Mm00437762_m1), *Ccr7* (Mm01301785_m1), *Cxcr5* (MM00432086_m1), *Foxp3* (Mm00475162_m1), *Gata3* (mm00484683_m1), *Gapdh* (Mm99999915_g1), *Icos* (Mm00497600_m1), *Ifng* (Mm01168134), *Il2* (Mm00434256_m1), *Il4* (Mm00445259_m1), *Il17a* (Mm00439618_m1), *Il21* (Mm00517640_m1), *Pdcd1* (MM00435532_m1), *Prdm1* (Mm01187285_m1), *Rorc* (Mm01261022_m1), *Tbx21* (MM00450960_m1), *Tnfrsf4* (Mm00442039_m1). Real-time PCR reactions were run in triplicate with an Applied Biosystems 7900HT Sequence Detection System (Life Technologies). Data were analyzed with SDS v2.3 software (Life Technologies), and normalized to the average of *Bact*, *B2m*, *Gapdh* expression.

### Statistical Analysis

GraphPad PRIZM V5 software (GraphPad Software, La Jolla, CA) was used for all statistical tests. Statistical significance was evaluated using an unpaired Student t-test with a 95% confidence interval. A P value of <0.05 was considered statistically significant.

## Results

### After protein immunization, a subset of CD4+CXCR5^high^ cells with a Tfh phenotype can be identified in the draining lymph nodes of BALB/c mice

Because the majority of murine Tfh studies have been conducted using C57BL/6 or B10.BR mice [8], [12]–[14], [51]–[55], we first evaluated previously established Tfh phenotypic markers in BALB/c mice. Eight days after immunization with the maltose binding protein from *E. coli* (MalE), a prototypic foreign antigen, emulsified in IFA with LPS, CD4+CXCR5^high^BCL6+ cells from the draining lymph nodes of BALB/c mice were evaluated by flow cytometry for the expression of markers used to identify Tfh cells in other strains of mice. Figure 1A shows that the expression of PD1 was equivalent to ICOS for the identification of CD4+CXCR5^high^BCL6+ cells. Additionally, these cells expressed uniformly high levels of CD44 (Fig. 1B). Figure 1D shows the expression of several Tfh associated markers on CD4+CD44^high^CXCR5^high^PD1^high^ (Tfh) cells and CD4+CD44^high^ cells that lacked high expression of either PD1 or CXCR5 (non-Tfh). Tfh cells were uniformly CD69+, ICOS+, BCL6+, CCR7^low^, and CD62L^low^, which is consistent with the phenotype of Tfh cells observed in other mouse strains.

Using these markers, we evaluated the kinetics of the Tfh and non-Tfh response in the draining lymph nodes. These distinct populations were evaluated by flow cytometry at days 0, 8, 15, and 22 after subcutaneous immunization with MalE protein. Both the Tfh and non-Tfh responses were robust by day 8 and contracted at least through day 22 post immunization (Fig. 1E). In subsequent experiments, unless stated otherwise, CD4 T cell responses were evaluated at day 8 or 9.

**Figure 2:**
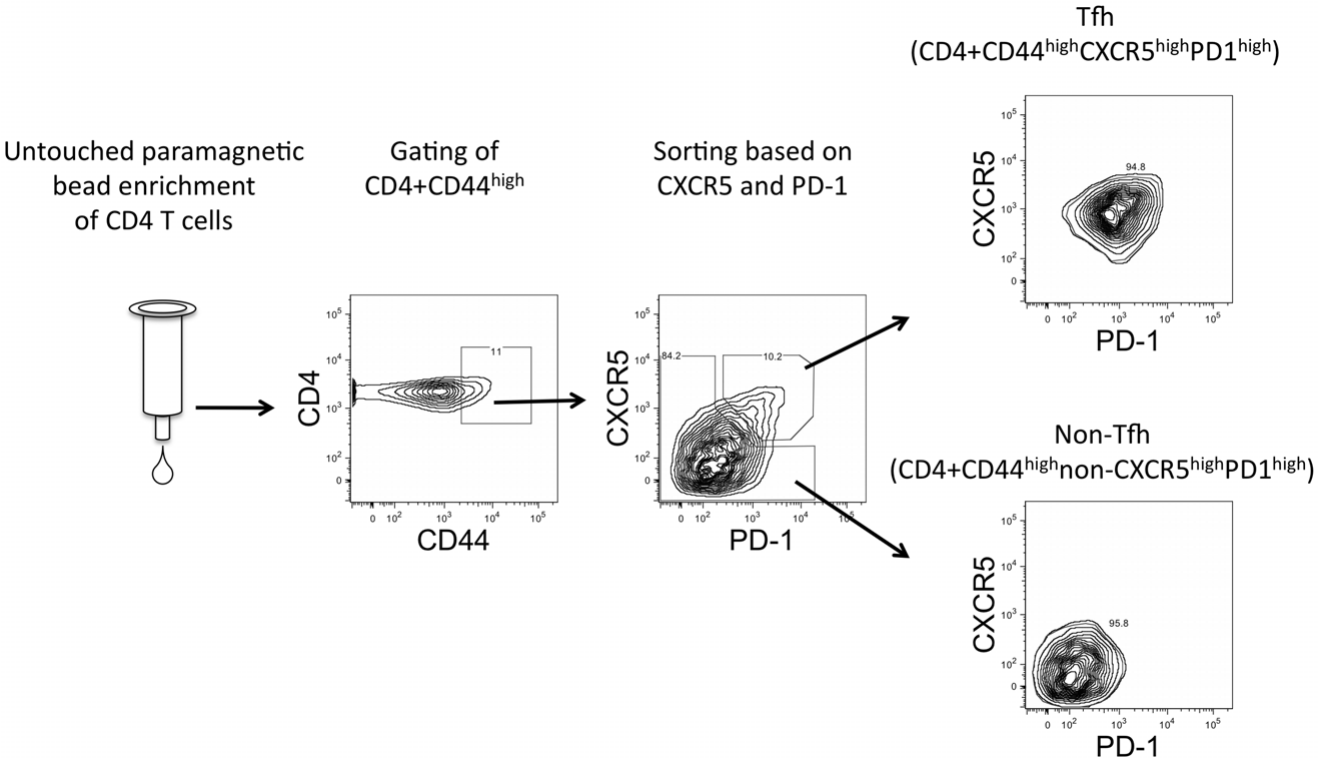
Schematic of sorting strategy used to separate Tfh from non-Tfh cells. CD4+ T cells are first enriched from draining lymph node cells with untouched paramagnetic bead separation. The CD4+ T cell enriched population is then stained for CD4, CD44, CXCR5, and PD1 and cytometry is used to sort stained cells by first gating on the CD4+CD44^high^ population and then separating Tfh from non-Tfh cells. Values displayed on plots are the percent of the population within each gate and are indicative of a representative experiment.

**Figure 3:**
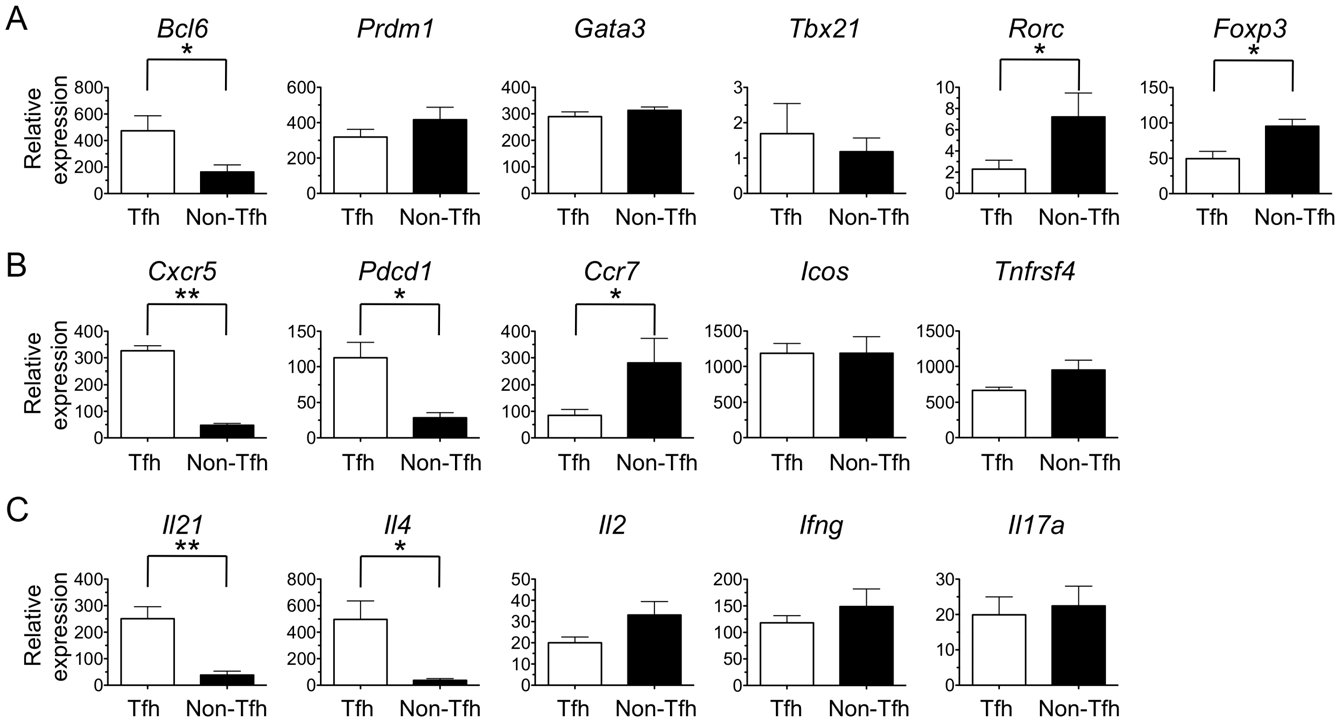
Sorted CD4+CD44^high^CXCR5^high^PD1^high^ cells express a transcriptional profile that is consistent with the Tfh phenotype. Real-time PCR analyses of cDNA synthesized from total RNA extracted from sorted Tfh and non-Tfh cells (as depicted in Fig. 2) and analyzed for expression of mRNA encoding transcription factors (A), chemokine receptors or accessory molecules (B), or cytokines (C). Expression data is normalized to the average expression of *Bact, Gapdh, B2m*. The means of four experiments are shown, with error bars indicating the standard error of the mean (S.E.M). Data were analyzed with unpaired two-tailed student T-test, and * indicates a p-value of less than 0.1, while ** indicates a p-value of less than 0.001.

To evaluate the peptide specificity of Tfh cells, a method was developed to separate endogenous polyclonal Tfh cells from antigen-experienced non-Tfh cells, as outlined in Figure 2. Briefly, total draining lymph node cells were enriched for CD4+ cells using negative paramagnetic bead selection, stained for CD4, CD44, CXCR5, and PD1, and then the CD4+CD44^high^ population was fractionated into “Tfh” (CXCR5^high^PD1^high^) and “non-Tfh” (CXCR5−/PD1−) populations by FACS. After cell sorting, the gene expression of the transcriptional regulators of T cell lineages (Fig. 3A), accessory molecules associated with Tfh cells (Fig. 3B), and several cytokines with lineage-associated expression (Fig. 3C) were evaluated in both cell populations. The transcriptional regulator of the Tfh lineage, *Bcl6*, the hallmark transcription factor associated with Tfh, is expressed at higher levels in Tfh population than in the non-Tfh population (Fig. 3A). The chemokine receptor expression pattern responsible for localization to the B cell follicle, *Cxcr5^high^Ccr7^low^*, is expressed within the Tfh, but not by the non-Tfh population (Fig. 3B), as expected. The basal level of Tfh-associated cytokines, *Il21* and *Il4*, are also more highly expressed in the Tfh population as compared to the non-Tfh population (Fig. 3C). Together, these data suggest that CD4+CD44^high^CXCR5^high^PD1^high^ cells in the draining lymph nodes of protein-immunized BALB/c mice, as in C57BL/6 and B10.BR, are indeed Tfh cells.

### The antigen specificity of Tfh cells can be measured with diverse cytokine-specific ELISPOT assays

**Figure 4:**
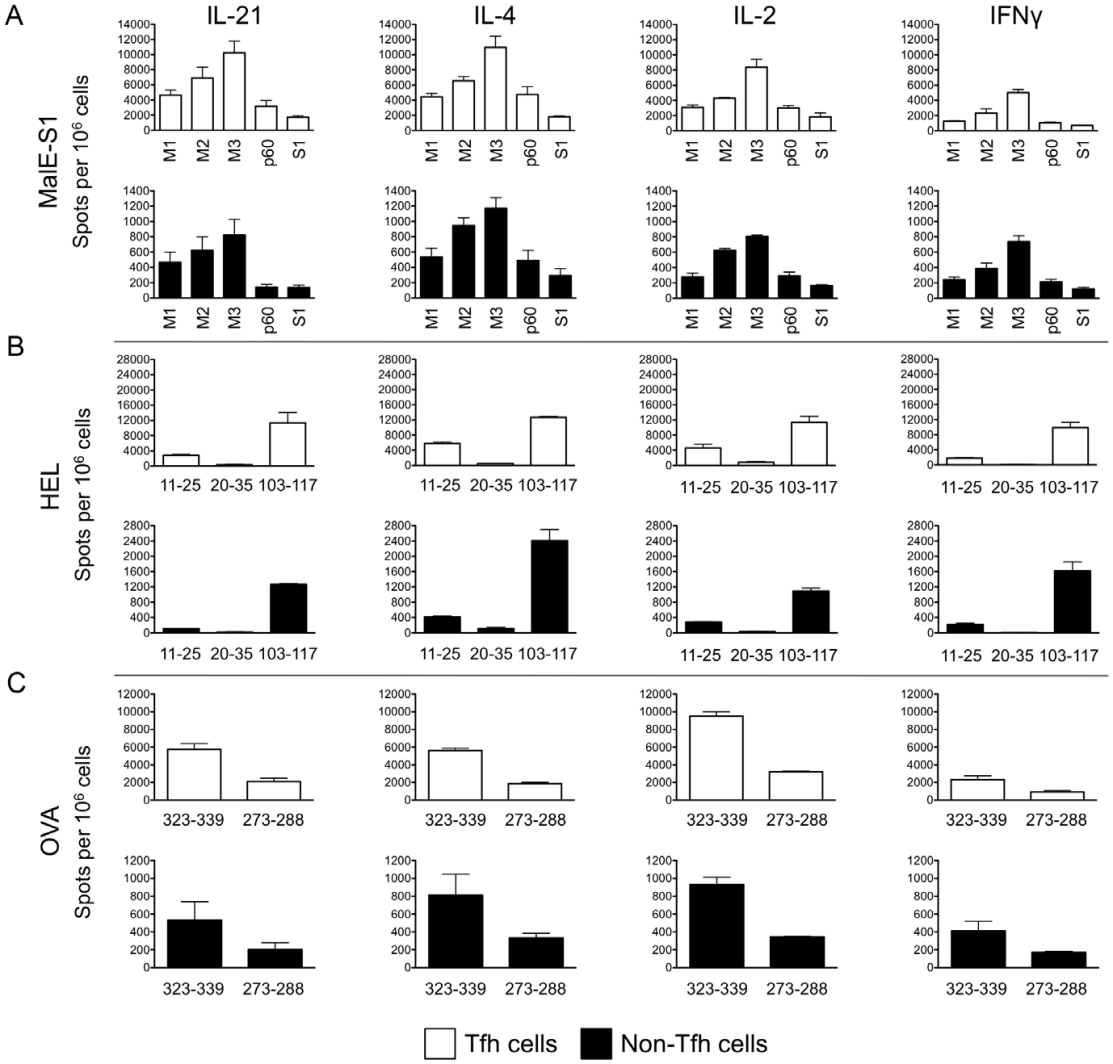
Peptide-specific Tfh responses can be measured with ELISPOT assays. On day 8 or 9 days post-immunization with MalE containing the HA-derived S1 epitope (A), HEL (B), or OVA (C), Tfh and non-Tfh cells were separated and peptide-specific responses were measured with IL-21, IL-4, IL-2 and IFNγ ELISPOT assays. The frequency of peptide-specific cytokine-secreting Tfh and non-Tfh cells are shown as cytokine-specific spots per 1,000,000 cells. Open and shaded bars show Tfh and non-Tfh cells respectively. The means of two experiments are shown, with error bars indicating the range.

While it is clear that Tfh cells can produce many cytokines in biologically relevant quantities [28], [56]–[58], there is some controversy regarding the amount of cytokine that is produced by Tfh cells compared to conventional CD4 T cells, by both intracellular cytokine staining [16], [28] and quantitative PCR [16], [53], [54], [59] approaches. Thus, it was not clear at the onset of this study if the peptide specificity of Tfh cells from the endogenous repertoire could be measured by cytokine-specific ELISPOT assays, after simulation of Tfh with peptide-bearing APC. To evaluate Tfh responses, cytokine-specific ELISPOT assays were employed because these assays are more sensitive than intracellular cytokine staining for the detection of rare epitope-specific CD4 T cells cells from the polyclonal endogenous T cell repertoire [60]–[62]. Our group has extensive experience using these assays for the measurement of epitope-specific responses [44], [45], [49], [50], [63]. To prevent potential bias in cytokine production between Tfh and non-Tfh populations, IL-21, IL-4, IL-2, and IFNγ secretion was measured for each population. Eight to nine days after mice were immunized with MalE, HEL, or OVA protein, the draining lymph nodes were excised, both Tfh and non-Tfh cells were isolated, as depicted in Fig. 2, and peptide-specific responses were evaluated. Detailed information on the antigenic peptides used for restimulation in ELISPOT assays are given in Table 1. Peptide-specific cytokine-secreting cells could be measured from the endogenous polyclonal Tfh and non-Tfh populations for all cytokines assessed (Fig. 4). The relative enrichment of cytokine-secreting cells in the Tfh population compared to the non-Tfh population may reflect an increased fraction of Tregs in the non-Tfh population, as suggested by increased *Foxp3* transcripts (see Fig. 3A).

**Table 1:** Antigenic peptides of model antigens.

Peptide	Source organism	Source protein	Sequence	MHC class II restriction
M1	*E. coli*	MalE 269-285	AKEFLENYLLTDEGLEA	I-A^d^
M2	*E. coli*	MalE 102-115	KLIAYPIAVEALSL	I-A^d^
M3	*E. coli*	MalE 69-84	GYAQSGLLAEITPDKA	I-A^d^
p60	*E. coli*	MalE 178-192	QEPYFTWPLIAADGG	I-A^d^
S1	*A/PR/8/34 Influenza*	HA 110-120	SFERFEIFPKE	I-E^d^
HEL 11-25	*G. gallus*	HEL 11-25	AMKRHGLDNYRGYSL	I-A^d^
HEL 20-35	*G. gallus*	HEL 20-35	YRGYSLGNWVCAAKFE	I-A^d^
HEL 103-117	*G. gallus*	HEL103-117	NGMNAWVAWRNRCKG	I-E^d^
OVA 323-339	*G. gallus*	OVA 323-339	KISQAVHAAHAEINEAGR	I-A^d^
OVA 273-288	*G. gallus*	OVA 273-288	MEERKIKVYLPRMKME	I-A^d^

**Figure 5:**
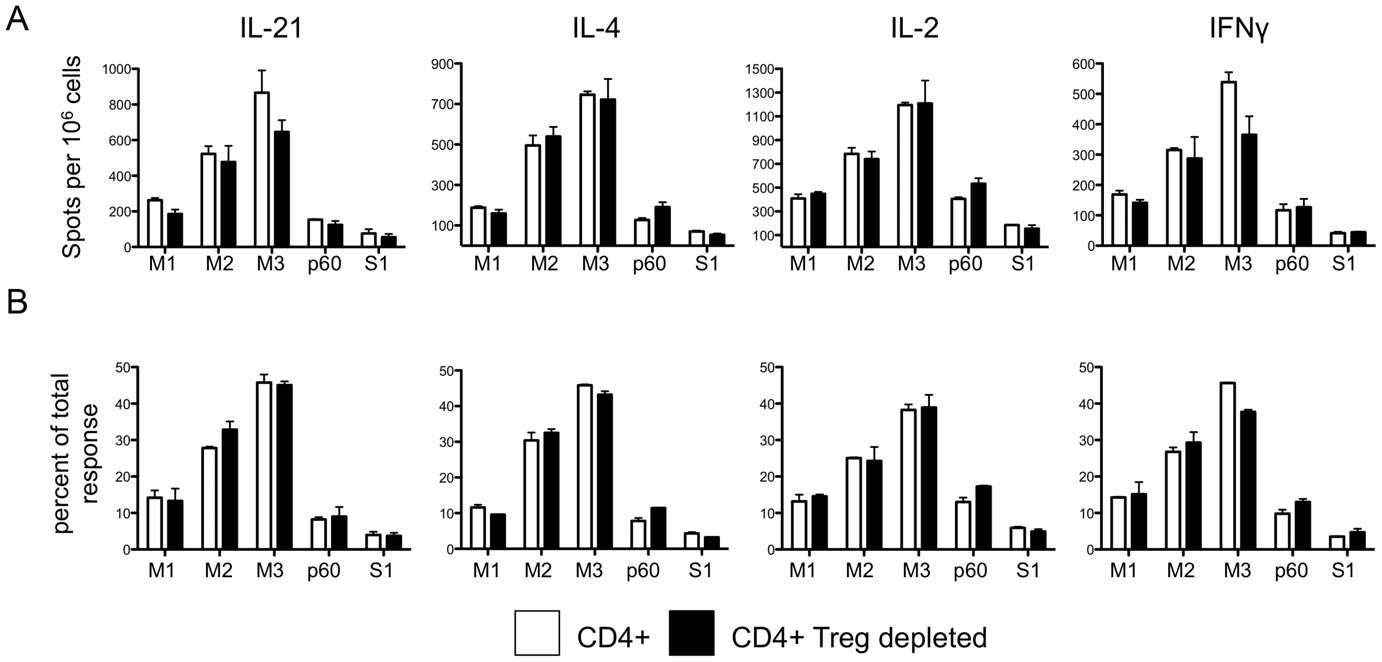
The frequency of cytokine secreting cells and the immunodominance hierarchies are not affected by the depletion of Tregs prior to ELISPOT assays. CD4 T cells were enriched from the cLNs on day nine after ear immunization with MalE-S1 protein. An aliquot of CD4 T cells was depleted of Tregs with CD25 microbeads prior to cytokine-specific ELISpot assays. Treg depletion (closed bars) did not increase the frequency of cytokine production (A) or alter the immunodominance hierarchies (B). Data are represented as the mean of two experiments with error bars depicting the range between experiments.

**Figure 6:**
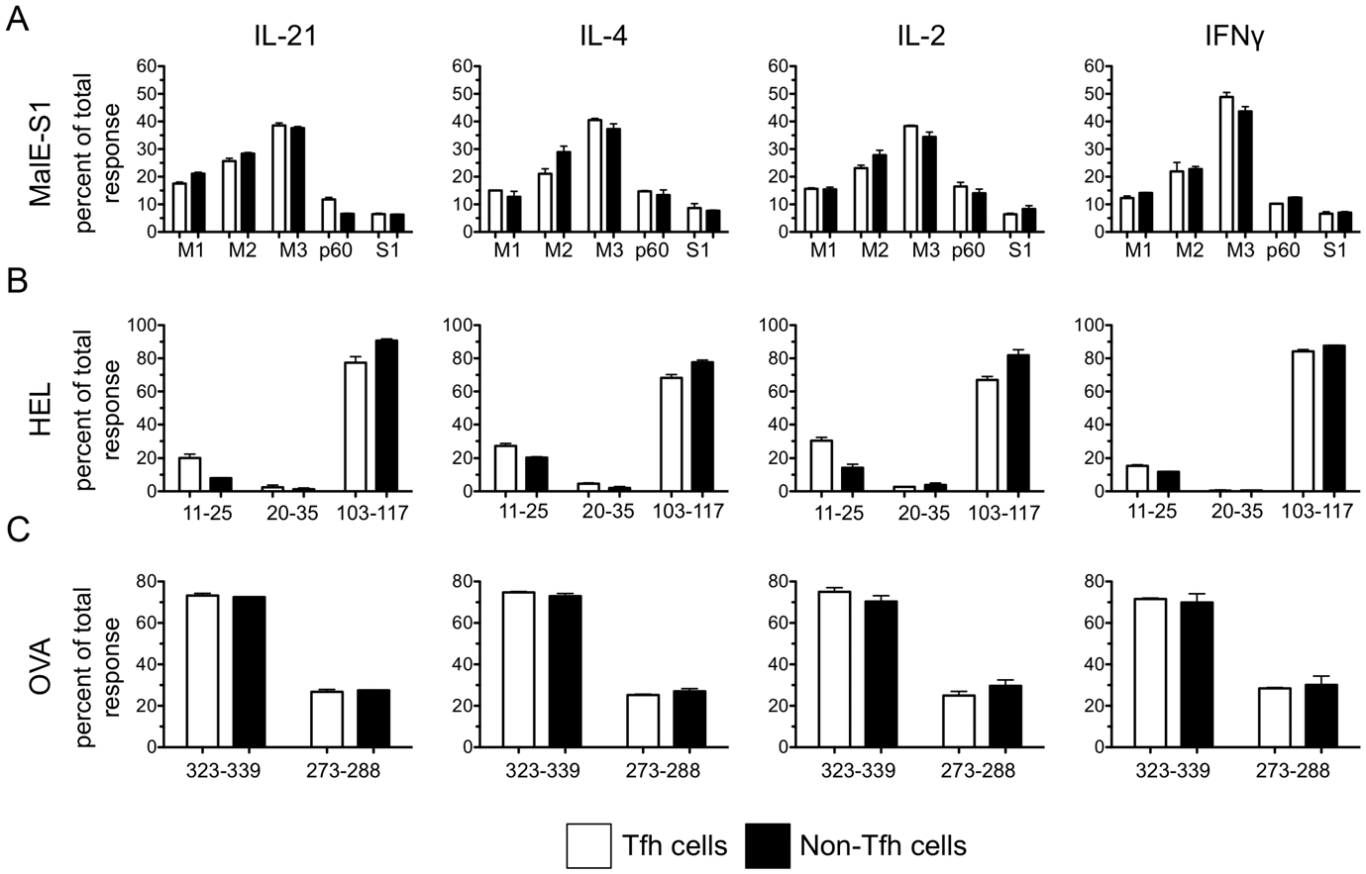
The peptide-specific hierarchies of the Tfh and non-Tfh population are remarkably similar, Independent of the cytokine assayed. The peptide specificities of Tfh and non-Tfh cells were evaluated with IL-2, IL-21, IL-4, and IFNγ ELISPOT assays on day 8 or 9 post-immunization. The percent of the total response for each peptide tested (peptide-specific/sum of all peptide responses measured) generated after immunization with MalE encoding the S1 epitope (A), HEL (B), or OVA (C) are shown for both Tfh (open bars) and non-Tfh (shaded bars). Data are presented as the mean of two experiments with error bars representing the range.

**Figure 7:**
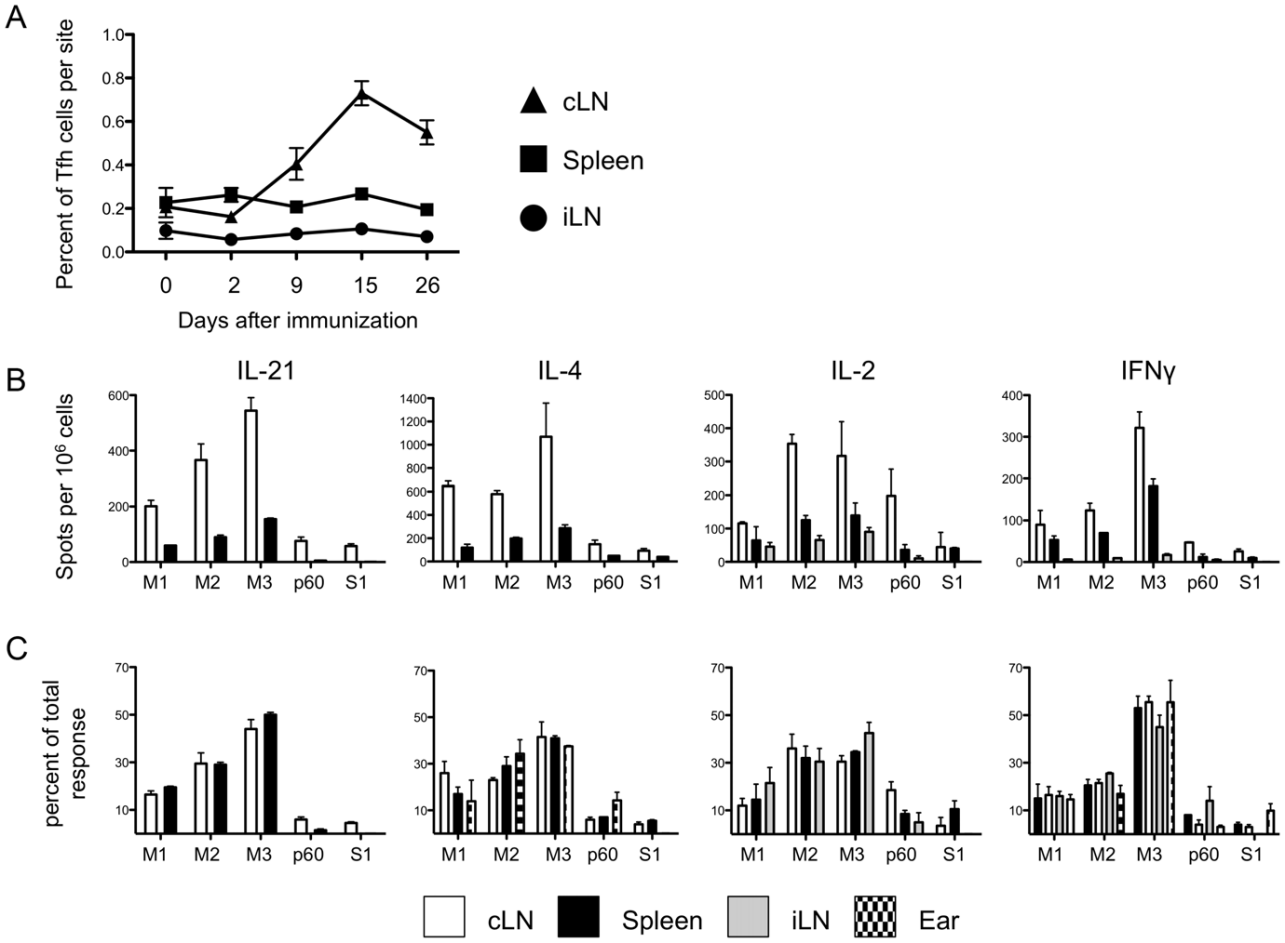
Immunodominance hierarchies established within the draining lymph node are maintained at secondary lymph tissues and at the site of immunization. The number of Tfh cells, as defined by CD4+CD44^high^CXCR5^high^PD1^high^ cells, enumerated from the cLN, iLN, spleen, or the ear are shown from unimmunized mice or mice immunized 2, 9, 15, or 26 days prior with MalE-S1 protein. The fraction of Tfh cells present at each site is shown, with the standard error of the mean (S.E.M) from four mice per time point (A). CD4 T cells were enriched from cervical and inguinal lymph nodes, as well as from spleen and the pinna of the ear 15 days after immunization of mice with MalE-S1 protein. The frequency of peptide-specific CD4 T cells was measured with ELISPOT assays and is shown in B while the percent of the total measured response is depicted in C. Frequencies are not given for ear samples due to very low responses (∼200 spots per ear). Responses from cells isolated from ear pinna were measured for IL-4 and IFNγ, while CD4 T cells from iLN were evaluated IL-2 and IFNγ production. Data represents the mean of two experiments with error bars represent the range.

**Figure 8:**
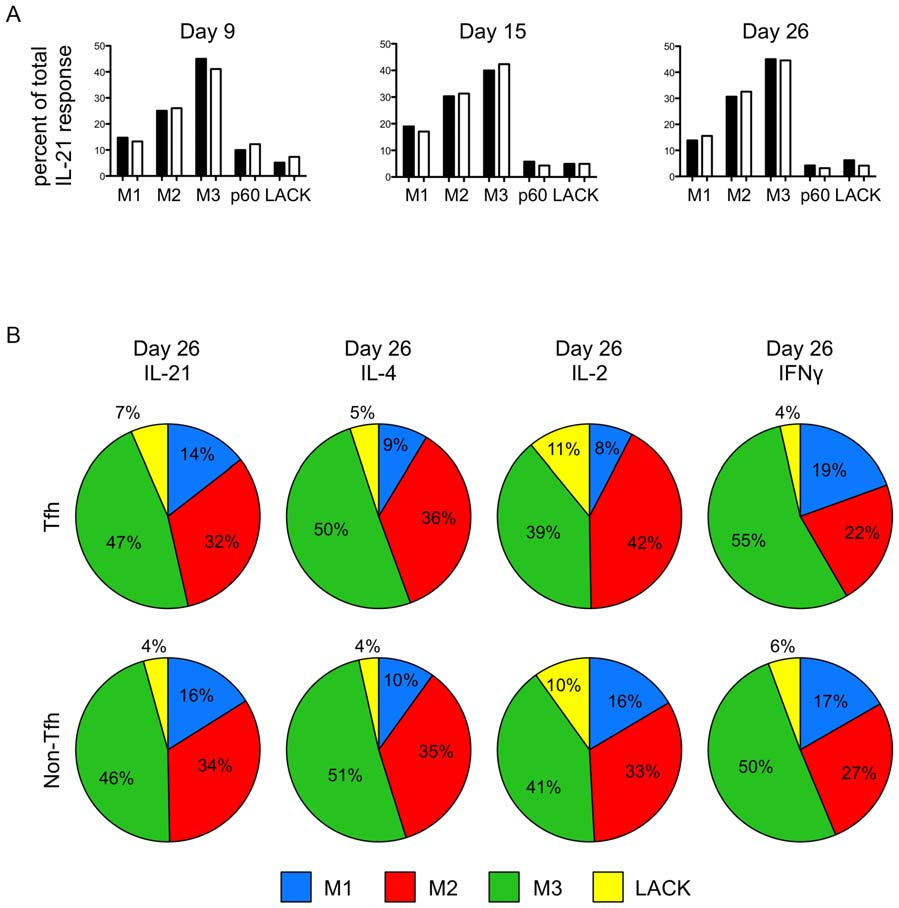
The immunodominance hierarchies established within the Tfh and non-Tfh populations during the peak of the immune response are maintained late in the immune response. As previously described, peptide-specific Tfh and non-Tfh cells responses were measured with IL-21 ELISPOT assays on day 9, 15 and 26 after MalE-LACK(I>A) protein immunization (A). On day 26 after protein immunization, peptide-specific Tfh and non-Tfh responses were measured with IL-21, IL-4, IL-2, and IFNγ ELISPOT assays. The percent of peptide responses are shown in B. Data shown represent a single experiment from cells pooled from 40–80 mice per time point.

**Figure 9:**
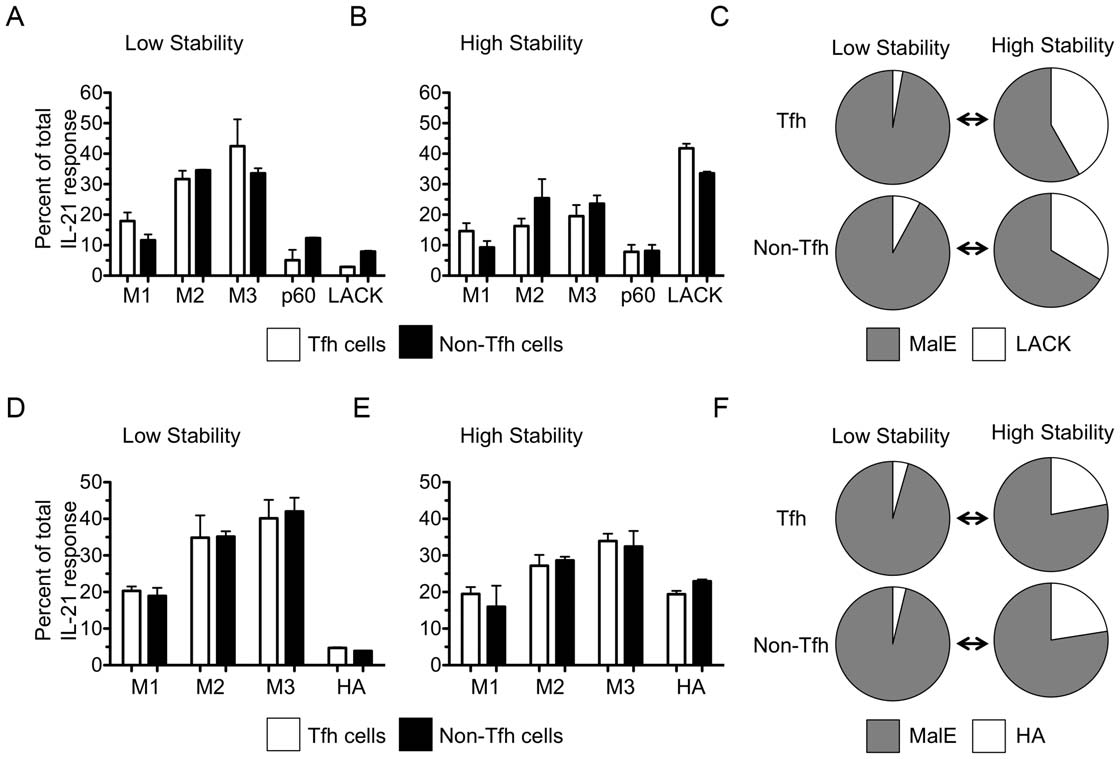
The kinetic stability of peptide∶MHC class II complexes determines the immunodominance of both Tfh and non-Tfh responses. Mice were immunized with MalE protein expressing either a high or low kinetic stability peptide variant. The persistence of peptide variants with MHC class II molecules are shown in Table 2. Two pairs (LACK and HA) of kinetic stability variants were evaluated and 20–25 mice were used per group. LACK responses are shown in the top row (A, B, and C) and HA responses are shown in the bottom row (D, E, and F). The left column (A and D) shows the percent of the IL-21 secreting Tfh (open bars) or non-Tfh (shaded bars) recalled after immunizing mice with the MalE protein bearing the low stability variant (LACK(WT) or HA(T>V)), while the center column (B and E) shows the percent of response recalled after immunizing mice with the MalE protein bearing the high stability variant (LACK(I>A) or HA(T>G)). The right column (C and F) depicts the fractional response of peptide variant (white) and sum of endogenous MalE peptides (gray) of either the Tfh or non-Tfh response. Data are shown as the mean of two experiments with error bars representing the range.

Shown are the antigenic peptides from maltose binding protein (MalE), hemagglutinin (HA), hen egg lysozyme (HEL), ovalbumin (OVA) that were used with APC to restimulate T cells *ex vivo* in cytokine-specific ELISPOT assays in Figures 4 to 9.

To test the contribution of Tregs, CD4 T cells were depleted of Tregs by CD25 negative paramagnetic microbeads selection prior to entry into cytokine-specific ELISPOT assays (Fig. 5). The depletion of Tregs did not affect the frequency of peptide-specific cells (Fig. 5A) detected by cyokine secretion or the peptide specificity of cells recalled (Fig. 5B). We think it likely that the apparent enrichment of Tfh cells for cytokine production reflects the fact that the non-Tfh population is partly composed of antigen-experienced memory cells from previous exposures to environmental antigens. Even in naive animals (day 0), a large fraction of CD44^hi^ T cells (8–10%) are present in the CD4 population. These irrelevant CD4 T cells would thus comprise a fraction of the “antigen-experienced” CXCR5^−^/PD1^−^ cells isolated in our studies, and thus dilute the apparent frequency of peptide-specific cytokine-producing CD4 T cells measured in the non-Tfh cells, relative to the Tfh population.

### The immunodominance hierarchy of the Tfh cell population generated in response to protein antigen strikingly parallels the hierarchy of the non-Tfh cell population

B cell antigen presentation is required for the maintenance of the Tfh lineage after the first few days of immunization (reviewed in [21], [22]), and therefore the set of peptides presented by B cells is likely a major selective factor that determines Tfh peptide specificity. Because B cells and DC have been suspected and shown (under some conditions) to present different repertoires of peptide-MHC class II complexes [29], [30], we reasoned that the immunodominance hierarchy of Tfh cells might be distinct from that of conventional CD4+ effector cells. To compare Tfh to non-Tfh immunodominance hierarchies, peptide-specific responses after MalE (Fig. 6A), HEL (Fig. 6B), or OVA protein (Fig. 6C) immunization, are represented as the percentage of the total response. These studies revealed that Tfh and non-Tfh populations shared nearly identical ranking of epitopes within the immunodominance hierarchy, independent of the cytokines used to measure responses. One epitope from MalE (p60: IL-21) and HEL (HEL 11–25: IL-21, IL-2, and IL-4) showed a slight but significant (P value of <0.05) increase in representation within the Tfh population compared to the non-Tfh population. Overall, these studies with independent antigens and multiple cytokines revealed that in polyclonal responses by the endogenous CD4 repertoire to foreign antigens, the follicular helper cell peptide specificity mirrors that of non-Tfh cells, destined to become either recirculating memory cells or effector cells. Even when extremely stringent gates were used to isolate Tfh cells, selecting only the brightest PD1+, CXCR5+ cells, leading to a 3–4 fold decrease in the yield of Tfh, the immunodominance hierarchy in the two populations was not changed (data not shown).

### Immunodominance hierarchies of CD4 T cells are maintained over time and at distal sites

It is known that after initial CD4 T cell proliferation driven by antigen-bearing dendritic cells, Tfh cells have prolonged opportunities to interact with B cells, while non-Tfh cells leave the lymph node [54], [64]–[66]. T cells that leave the lymph node either home to the tissue site of antigen exposure or enter the long lived recirculating pool of memory cells (reviewed in [67]–[71]). To address whether immunodominance hierarchies in the draining lymph node change over time or whether cells that leave the lymph node display different patterns of immunodominance from those within the lymph node, we analyzed CD4 T cell specificity over time and in different tissues. Figure 7A shows the frequency of Tfh cells over time, using the markers CD44^high^, PD1^bright^, CXCR5^bright^. As expected, within the draining cervical lymph node (cLN), Tfh expand early and then begin to diminish between day 15 and day 26 post-vaccination. At no time point tested was there any gain in cells with Tfh makers in the spleen or non-draining inguinal lymph node (iLN), where they comprise <1% of the total population of cells. Additionally, no Tfh cells were detected at the site of immunization (not shown). To examine the specificity of the response at different sites, CD4 T cells from draining and non-draining lymph nodes, spleen and site of immunization were tested for reactivity with the individual peptides contained in the MalE antigen (Figure 7B and 7C), using cytokine ELISPOTS assays. While the frequency of peptide-specific cells was reduced in distal tissues (Fig. 7B), no differences in immunodominance were noted (Fig. 7C). Figure 8 shows the immunodominance hierarchy from Tfh and non-Tfh over time (day 9, day 15 and day 26) from the draining lymph node, again testing for each of the four cytokines at day 26 (Figure 7B) and multiple peptides at each time point. These studies revealed that the immunodominance hierarchies detected early and in the draining lymph node persist over time and in the re-circulating pool of CD4 T cells.

### Increasing the persistence of peptide-MHC class II complexes enhances epitope-specific generation of Tfh cells

Studies by our group have shown that the persistence of a peptide with MHC class II molecules is a key feature in determining its immunodominance in the elicited response [48], [50], [72], [73]. If this deterministic feature extends to Tfh cells, it may be possible to apply this relationship to the design of epitope-based vaccines to focus the Tfh response. To study the effect of peptide persistence on the Tfh immunodominance hierarchy, mice were immunized with MalE proteins encoding a kinetic stability variant peptide that had altered MHC anchor residues leading to variations in off-rates from MHC class II. Variants were chosen that persist for long (over 70 hours) or short (under 15 hours) periods of time with I-A^d^ (details about kinetic stability peptide variants are shown in Table 2 and have been published previously [48], [50], [72]). Because drainage of antigen could be continuous after vaccination with the oil-in-water emulsions used in our experiments, the site of antigen depot (ear pinna) was excised three days post-immunization to generate a more synchronous cohort of APC to initiate the immune response.

**Table 2:** Kinetic stability peptide variants.

Peptide	Source organism	Source protein	Sequence	MHC class II restriction	Peptide-MHC class II t_1/2_(h) pH 7.4
HA(T>V)	*A/PR/8/34 Influenza*	HA 126-138, T128V	HNVNGVTAASSHE	I-A^d^	160
HA(T>G)	*A/PR/8/34 Influenza*	HA 126-138, T128G	HNGNGVTAASSHE	I-A^d^	13
LACK(WT)	*L. major*	LACK 161-173	SLEHPIVVSGSWD	I-A^d^	70
LACK(I>A)	*L. major*	LACK 161-173, I166A	SLEHPAVVSGSWD	I-A^d^	1

Shown are the kinetic stability peptide variants from hemagglutinin (HA) and Leishmanina antigen protein kinase (LACK) evaluated in Figure 9.

IL-21 cytokine-specific ELISPOT assays were used to quantify peptide-specific responses from Tfh or non-Tfh cells isolated from draining lymph nodes at 9 days post immunization with MalE protein bearing the kinetic stability variant peptides (Fig. 9). The percent of the total Tfh or non-Tfh response recruited by each peptide epitope in response to MalE protein encoding the LACK (I<A) epitope (low stability) or LACK (WT) epitope (high stability) are shown in Figures 9A and 9B respectively. The fractional response to variant peptides and summed responses to MalE peptides are shown in Figure 9C. Similarly, responses to a second set of peptide variants, HA (T>G) epitope (low stability) and HA (T>V) epitope (high stability) are represented in Figures 9D and 9E, respectively, and Figure 9F. These experiments revealed that the generation and/or maintenance of both epitope-specific Tfh and non-Tfh cells were enhanced by priming with peptides that persist for longer periods with the MHC class II molecule (Fig. 9C and 9F). This result demonstrates that increasing the stability of peptide-MHC class II complexes bolsters the Tfh response, while destabilizing these complexes diminished the epitope-specific Tfh response. The ability to enhance, diminish, and focus the Tfh response in an epitope-specific manner could be a powerful tool for the rational design of next-generation vaccines.

## Discussion

These studies were initiated to characterize and compare the immunodominance hierarchies of the Tfh population generated from the endogenous CD4 T cell repertoire after protein immunization, an issue that has yet to be explored. The potential of these populations to produce cytokine in response to peptide-bearing APC was also experimentally evaluated. Overall, the studies described here indicate that Tfh cells within the lymph node are robust producers of many cytokines, peptide-specific responses can be evaluated with diverse cytokine-specific ELISPOT assays, and the immunodominance hierarchy of the peptide-specific responses within the Tfh and non-Tfh subsets are remarkably similar. Using these assays, we found that the immunodominance hierarchies of Tfh and non-Tfh populations were similar at the peak of the immune response, and persisted for over 25 days post immunization. At day 8–9 post immunization, two I-A^d^ restricted epitopes (p60 and HEL 11–25) had a slight but significant increase in the Tfh population, but were of low frequencies and therefore these slight changes may not reflect biologically relevant variability.

After priming, classical effector T cells egress from the lymph node and traffic to distal secondary lymphoid tissue or the site of pathogen challenge, while Tfh cells have been shown to persist for relatively long periods of time in the draining lymph node [65]. Thus, we considered the possibility that restricting our comparison of Tfh and non-Tfh to the population of cells in the lymph node may not permit full assessment of diversity of CD4 T cells generated during the immune response. To address this, first we verified that Tfh cells do not detectably accumulate in the distal tissues (spleen and iLN) or the site of immunization, at least through day 26 post immunization. Secondly, we found that epitope-specific distribution of the CD4 T cell response in the spleen closely parallel the distribution in the draining lymph node. Although B cells have distinct mechanisms of antigen uptake (reviewed in [28]), expression of proteases (reviewed in [34], [35]), and expression and localization of H-2O (reviewed in [34], [36]) compared to DC, our results suggest that after protein immunization, these differences are not sufficient to alter the amounts of individual peptides displayed by the antigen-specific B cell compartment in a way that limits further recruitment of Tfh cells. These results lead us to conclude that during responses by the endogenous repertoire to completely heterogonous antigens, it is DC priming, rather than antigen presentation by B cells that is likely the limiting factor for Tfh generation, and little “pruning” of CD4 T cell specificity takes place under the conditions of protein vaccination studied here.

We can envision several scenarios where the Tfh repertoire might be more distinct from the non-Tfh effector population of CD4 T cells. Included in these are conditions when the number or specificity of responding antigen-specific B cells is increased by repeated encounters by vaccination or when Ig-mediated uptake of antigen occurs by a restricted B cell repertoire, where Ig binding in close proximity with peptide epitopes could interfere or potentiate epitope-specific presentation by B cells [31], [32]. The impact of these parameters on modifying the Tfh repertoire is currently under investigation. The impact of altering peptide persistence on the immunodominance hierarchy of the unfractionated CD4 T cell post vaccination and infection has been previously described by our laboratory [48], [50], [72], [73]. The study here demonstrates that increasing or decreasing the stability of peptide-MHC class II complexes by changing residues in the MHC anchor positions directly influenced the immunodominance patterns of the Tfh compartment. Both Tfh and non-Tfh responses were bolstered when peptide persistence was increased, while conversely, decreasing the stability of peptide-MHC class II complexes diminished responses. We suspect that DC presentation mediates this effect because both Tfh and non-Tfh responses are coordinately modulated by this parameter. These results indicate that altering the kinetic stability of peptide binding to MHC class II is a method of directing or focusing the Tfh response. It will be important to evaluate the functional consequences of altering the Tfh response on the generation of protective antibody responses for next generation vaccine design.
